# Healthcare Needs and Perceptions of People Living With Inflammatory Bowel Disease in Australia: A Mixed-Methods Study

**DOI:** 10.1093/crocol/otab084

**Published:** 2022-01-03

**Authors:** Sharmila S Prasad, Marjorie M Walker, Nicholas J Talley, Simon Keely, Therése Kairuz, Michael P Jones, Kerith Duncanson

**Affiliations:** 1 School of Biomedical Sciences and Pharmacy, College of Health, Medicine and Wellbeing, University of Newcastle, Callaghan, New South Wales, Australia; 2 NHMRC Centre of Research Excellence in Digestive Health, University of Newcastle, New Lambton Heights, New South Wales, Australia; 3 School of Medicine and Public Health, College of Health, Medicine and Wellbeing, University of Newcastle, New Lambton Heights, New South Wales, Australia; 4 School of Psychological Sciences, Macquarie University, Macquarie Park, Sydney, New South Wales, Australia; 5 School of Health Sciences, College of Health, Medicine and Wellbeing, University of Newcastle, New Lambton Heights, New South Wales, Australia

**Keywords:** inflammatory bowel disease (IBD), healthcare professionals, patient perception

## Abstract

**Background:**

Crohn’s disease (CD), ulcerative colitis (UC), and indeterminate colitis are inflammatory bowel diseases (IBDs) that adversely affect the healthcare needs and quality of life (QoL) of people with IBD. The aim of this study was to explore the needs and perceptions of people with IBD in a primary care setting.

**Methods:**

This sequential explanatory mixed-methods study consisted of a cross-sectional survey (included validated tools), followed by semistructured interviews on participants’ perceptions: IBD management, healthcare professionals, IBD care, flare management, and pharmacist’s IBD roles.

**Results:**

Sixty-seven participants completed the survey, and 8 completed interviews. *Quantitative findings*: Age at diagnosis had significant association with medication nonadherence (*P* = .04), QoL (*P* = .04), and disease control (*P* = .01) among the respondents. The odds of medication nonadherence were 8 times (adjusted odds ratio [AOR] = 8.04, 95% confidence interval [CI] = 1.08, 60.10) higher among younger participants aged <30 years. Those diagnosed with CD (*P* = .02) reported more likely to have unfavorable perceptions of pharmacists' role in managing their IBD (AOR = 9.45, 95% CI = 1.57, 56.62) than those with UC and indeterminate colitis. *Qualitative findings*: General practitioners were considered the most important care provider and the first point of contact for patients in managing all aspects of IBD. Participants identified their key need to be timely access to specialized IBD care and found that other primary healthcare professionals lacked disease-specific knowledge for managing IBD.

**Conclusions:**

Primary healthcare professionals are well positioned but need targeted training to influence the needs of IBD patients. The specialty role of an IBD educator could complement existing services to deliver and address patient-specific care.

## Introduction

Inflammatory bowel diseases (IBDs) are chronic inflammatory conditions affecting the gastrointestinal tract that include Crohn’s disease (CD), ulcerative colitis (UC), and indeterminate colitis.^1^ The burden of IBD is rising globally, with prevalence estimated to exceed 0.3% in North America, Oceania, and many European countries. IBD incidence is increasing in developing and newly industrialized countries.^1,2^ The estimated prevalence of IBD in Australia is 0.4% and the number of Australians with IBD is expected to rise dramatically over the next 10 years.^2^ Australia has among the highest IBD incidence in the world, with relatively more young people (aged 15–40 years) being diagnosed each year.^1,2^

IBD generally has an early life onset, is associated with considerable morbidity and an unpredictable relapsing and remitting disease course.^1,3^ The symptoms experienced by people with active disease negatively impact their physical, social, and psychological wellbeing.^4^ These include severe abdominal pain, loss of bowel control, frequent bloody diarrhea, weight loss, fatigue, and anxiety or depression. Illness-related loss of employment and limited social interactions also contribute to a reduced quality of life (QoL).^5^ IBD is also associated with complications that have serious consequences, including hospitalizations, multiple surgeries, disease complications, and increased risk of developing colorectal cancer.^6^

IBD patients generally require ongoing treatment with medications to maintain disease remission.^1,5^ Even when in remission, IBD continues to negatively influence patients’ needs, experience, and outlook on life.^7^ Studies have shown that increased pain perception among people living with IBD is associated with anxiety and depression,^8,9^ and disease flares are linked with poor QoL.^7^ Disease severity has been linked to psychological distress that resulted in poor coping^4,8^ and fear of medication-related adverse effects have been associated with nonadherence.^10,11^ The need for long-term use of medical therapies to maintain disease remission, therefore, creates other obstacles for patients.^12^

Gaps in care can contribute to a greater burden of disease to individuals, healthcare professionals, and the healthcare system.^1,2^ In Australia, patients with IBD are predominantly managed by gastroenterologists in secondary and tertiary care settings.^6^ The Australian IBD Audit of hospital care, and other published literature have reported inconsistent or variable IBD patient care that fails to meet national standards.^1,13^ A model of care based exclusively on specialist care may lead to considerable barriers to timely access to healthcare and multidisciplinary services, provision of educational information and resources, compounding the gaps in care and unmet needs of patients living with IBD.^1,6^ There has not been an equivalent audit of IBD primary care, but current IBD standards suggest a multidisciplinary team structure as an ideal approach to managing patients with IBD.^14^

Promoting patient engagement is an important part of chronic disease management.^15^ In IBD, patient involvement and active participation in decision-making have been positively reported with clinical effectiveness and improved patient-related health outcomes.^16^ Despite the complexity of IBD etiology and management, the extent and nature of healthcare needs among patients with IBD are not widely understood.^12^ Understanding factors that affect the needs of these patients could be an important step toward optimizing patient-centered IBD care.^12^ There is growing interest in patients’ perceptions of both their care and their engagement with healthcare professionals involved with their care.^17^ Patients’ views and expectations are as important to their care as the assessment of the quality of care and the delivery of care-related services.^17^

IBD patients are particularly affected by physical symptoms throughout their disease and tend to visit primary care providers (general practitioners [GPs] and pharmacists) as their first point of contact.^6,18^ In a recent publication, we explored how pharmacists perceived themselves and other primary healthcare professionals in managing patients with IBD.^19^ The current study focuses on the needs of IBD patients and the opportunities for primary care professions including pharmacists. To fully understand and to meet the needs of people with IBD using patient-centered care, the patient perspective is also required. The aim of this study was to: (1) assess patient-reported disease control, QoL, and extent of medication nonadherence in patients with IBD and, based on this assessment (2) use semistructured interviews to explain quantitative survey results and elicit patients’ views toward their engagement with healthcare professionals in a primary care setting.

## Materials and Methods

This explanatory mixed methodology included a quantitative survey followed by qualitative semistructured interviews. The study population was Australian adults living with IBD.

### Study Participants

From August 2019 to April 2020, patients with IBD were enrolled in a cross-sectional survey about their IBD-related needs and perceptions about healthcare. The study participants were recruited through the Digestive Health Biobank (DHB) Hunter Medical Research Institute, Newcastle NSW and through the national not-for-profit organization, Crohn’s & Colitis Australia. Inclusion criteria were: a clinical diagnosis of IBD; willingness to consent to participation; at least 18 years of age; and ability to understand the questions and communicate in English. People with gastrointestinal tract malignancies (eg, colorectal cancer) were not eligible to participate, due to their substantially different healthcare needs.

To ensure a statistically reliable estimate, a sample size available for analysis was calculated using a pragmatic approach based on the number of participants in studies that reported clinically relevant changes in HRQoL using the validated Short Inflammatory Bowel Disease Questionnaire (SIBDQ).^20–22^ The required analysis sample size based on the correlation coefficient between HRQoL and SIBDQ from previous studies of 0.3,^20,22^ was estimated at 85 people with IBD (significance level of 0.05 and power of 80%).

Participants from the DHB were sent a letter of invitation, the survey and a reply-paid envelope; which also included a link to allow the participant the choice of completing the survey online. Participants from Crohn’s Colitis Australia were able to access a link to the online survey on their official website. Completion of the survey was deemed as consent for the study (see Supplementary Data 1, which includes participant information).

### Quantitative Study Design

An exploratory cross-sectional survey was developed using relevant literature,^1,3,23^ systematic reviews and available validated instruments relating to patients’ needs in chronic disease and IBD management. For face and content validity, questions were piloted among a convenience sample of 10 individuals (2 gastroenterologists, 6 research students, 1 GP, and a researcher in the field of IBD). Based on the results of this pilot testing, the final survey was created and printed for circulation. The online format was uploaded to a secure web application—Research Electronic Data Capture (REDCap), created in 2004 by Vanderbilt University, designed for clinical and translational survey research.

The survey comprised 6 sections and took approximately 30 minutes to complete. The information included demographics and questions seeking to understand patients’ needs relating to medication nonadherence, QoL, and IBD-control. A combination of binary, multiple-choice, free-text responses, 5- and 7-point Likert scales, and ranking format were used (Supplementary Data 1, which includes participant information).

### Survey Measures

For the assessment of IBD-control, our survey included a modified version of the validated 10-item IBD Control Questionnaire.^24,25^ Instead of scoring high IBD-control as “good” disease control, the survey included IBD-control-VAS ranging from 0 (worst control) to 100 (best control) for each question. An overall sum score was calculated with a cutoff for the remission of ≥8, consistent with other published literature.^24,25^ Questions relating to patient’s next clinic visit were not incorporated in the survey as they were not relevant to this study. Whilst we did not validate the modifications among IBD patients, the survey was piloted for face and content validity among academics, researchers, and clinicians.

A target cutoff point of 75% was selected to define adherence from nonadherence. This cutoff is consistent with Zand et al,^26^ who reported 6 points out of 8 questions to categorize the respondent as “adherent.” In our cohort, 7.5 out of 10 points was the cutoff between adherent (>75%) and nonadherent (≤75%).

Patients’ QoL was measured using the SIBDQ. The SIBDQ is a 10-item disease-specific questionnaire,^27–29^ used to evaluate HRQoL in 4 dimensions: bowel symptoms, systemic symptoms, emotional functioning, and social functioning. Each question has a graded response ranging from 1 (poor quality) to 7 (better quality). Scores from individual domains are summed to obtain a total score ranging from 10 to 70, which corresponds with the overall poor-to-better QoL (QoL). A total score of 42 or lower was categorized as “poorer” QoL and above 42 as “better” QoL, consistent with published literature using the SIBDQ.^27,28,30–32^

Lack of literature defining patients’ perception about pharmacists’ role in IBD management and no established criteria, led us to use a statistical method to operationally define our outcome variable. Patients’ perceptions were categorized into unfavorable (<55) and favorable (≥55) using the median value of the overall perception score as a cutoff point. For rigor, the strengthening of the reporting of observational studies in epidemiology (STROBE) statement was used for the quantitative component of the study. (Supplementary Data 2, which describes the study rigor).^33^

### Survey Statistical Analysis

Descriptive statistics were used for study participants’ characteristics. To examine the association between participants’ background characteristics with medication nonadherence and QoL, Pearson’s chi-square and Fisher’s Exact Test were performed to include disproportionate marginal frequencies. The reliability test of the SIBDQ and the IBD Control Questionnaire showed high internal consistency with a Cronbach’s alpha of 0.91 (SIBDQ) and 0.89 (IBD-control). Possible relationships between disease (IBD) control score and participants’ characteristics were evaluated with Mann–Whitney–Wilcoxon test for 2 groups and Kruskal–Wallis test when comparisons involved more than 2 groups. Associations between quantitative variables were evaluated using the Spearman rank correlation. Pearson’s chi-square or Fisher’s exact test was performed to examine the statistical significance of differences in patients’ perception toward pharmacists’ role in managing IBDs.

Multiple logistic regression analysis was performed to identify statistically independent factors associated with medication nonadherence (age of the time of IBD diagnosis-control: ≥30 years; household status-control: living with people; medical conditions-control: no medical conditions), QoL (age of the time of IBD diagnosis-control: <30 years; gender-control: female; current management of IBD-control: injectables/biologics; side effects from IBD medications-control: no/unsure; complications associated with IBD-control: no complications), and disease control and to identify factors associated toward a negative perception of pharmacists’ role in managing IBD patients (current age-control: >40 years; type of IBD-control: indeterminate colitis/unsure). The odds ratio was used as a measure of association and is reported with a 95% confidence interval (CI). A *P* value of less than .05 was set for statistical significance. The reliability test of the patients’ perception assessment tool showed high internal consistency with a Cronbach’s alpha of 0.94. Analysis of the data was performed using Stata version 14 statistical software (StataCorp. Stata Statistical Software: Release 14. College Station, TX: StataCorp LP, 2015).

### Qualitative Study Design

Semistructured interview questions were developed from data generated during the analysis of the cross-sectional survey. The questions related to engagement with health professionals by people with IBD, with a focus on topics of disease control, QoL, extent of medication nonadherence, and primary care health professional engagement, with a particular focus on pharmacist engagement. A purposive sampling approach was used to recruit a subset of participants who had already completed the quantitative survey,^34^ and who indicated a willingness to participate in further research.

### Qualitative Data Collection

The interviews were conducted via videoconferencing or telephone by SP between December 2020 and February 2021. An interview question guide (Supplementary Data 3, which describes the questions) was designed to explore patients’ perceptions and facilitate expression of their perceptions in their own words. The interview guide was pilot tested and refined based on individual feedback from 2 independent qualitative researchers. Individual interviews were digitally recorded and professionally transcribed for analysis. Recordings were carefully checked for accuracy then destroyed to protect participants’ privacy.

### Qualitative Data Analysis

The Framework methodology informed qualitative data analysis.^35^ A draft analytic framework was developed, a priori (Supplementary Data 4), based on the findings of the cross-sectional survey and interview question topics. Word document transcripts were independently open coded by S.P. and K.D., with coding then compared and consolidated. The draft matrix was then populated with coded data by S.P., with coded data grouped together within the a priori themes. The framework was further developed and adapted as coded data were added, in parallel with discussions about data interpretation between S.P. and K.D.^35^ For the purpose of rigor, coding and themes were examined by a third reviewer (K.T.). The data in the resulting framework were triangulated with the quantitative data. The matrix output included references to interesting or illustrative quotes to exemplify code meanings. All reviewers agreed on the codes and themes and the consolidated criteria for reporting qualitative research tool (COREQ) were used to ensure rigor within the data analysis process (Supplementary Data 5, which describes the study).^36^

### Ethical Considerations

The study protocol was reviewed and approved by the Research Ethics Committee at Hunter New England Local Health District [2019/ETH00167] and the University of Newcastle Human Research Ethics Committee (HREC) [H-2019-0201] prior to commencement.

## Results

Detailed participant information is included in Supplementary Data 6.

### Factors Associated With Disease Control in Patients With IBD

Overall, 14 (69.3%) respondents reported to have well-controlled IBD, 13 (21%) did not have their IBD under control and 6 (9.7%) were unsure about their disease control. Based on the cutoff for remission of ≥85, only 9 (14.5%) respondents were in remission while 53 (85.5%) were not. Interestingly, 45 (72.6%) of the respondents did not think they needed a change to their treatment, 13 (21%) reported needing treatment change and 4 (6.4%) were unsure (Supplementary Data 7, which reports IBD-control characteristics). Age at the time of diagnosis (*P* = .01), IBD medication-related side effects (*P* = <.01), and complications associated with IBD (*P* = .03) (Supplementary Data 8, which describes participants association between characteristics and IBD-control) were independently associated with poor overall IBD-control.

Qualitative subthemes that related to disease control included management preferences, type, timing and accessibility of healthcare, and knowledge and communication by healthcare professionals. Several participants suggested that self-management efficacy was important in their control of IBD when they had symptoms. Some participants indicated seeking medical management as a preferred option for disease or symptom control, while others suggested that their disease control was best managed as a combination of medical care and self-management. In contrast, 2 participants prioritized multidisciplinary involvement in disease control and some participants felt it was imperative to have easy and timely access to medical services as essential to their disease management or control. For example:

It is access to specialists and more importantly, it’s the timing of it. You don’t want your specialist to get back to you in a week. A week is a long time for someone with IBD and a lot can go wrong in that time. So timely manner is so important. (Male, aged 56, diagnosed CD)

Participants reported knowledge and communication as important factors for optimizing their disease control, with self-learning and healthcare professionals’ knowledge and understanding of IBD considered equally important. Several participants reported the need for ongoing guidance through the course of their disease.

### Factors Associated With Medication Nonadherence in Patients With IBD

Based on our target cutoff point of 75% to define adherence, 58 (86.6%) respondents were adherent to their medication and 9 (13.4%) were nonadherent. Medication nonadherence was associated with age at diagnosis of the disease (*P* = .04), household status (*P* = .004), and having other medical conditions (*P* = .02) (Supplementary Data 9, which reports factors associated with medication nonadherence). The odds of medication nonadherence among IBD participants aged less than 30 years at the time of IBD diagnosis were significantly higher (adjusted odds ratio [AOR] = 8.04, 95% CI = 1.08, 60.10) compared with those aged over 30 at the time of their IBD diagnosis. Similarly, the odds of medication nonadherence were significantly much higher (AOR = 19.37, 95% CI = 2.37, 158.28) among participants who lived alone compared with those who lived with other people (Table 1).

**Table 1.** A multivariable logistic regression of factors associated with medication adherence.

Variables	Medication adherence		AOR (95% CI)
	Adherent	Nonadherent	
Age at the time of IBD diagnosis (years)			
<30	24 (41.4)	7 (77.8)	8.04 (1.08, 60.10)
≥30	34 (58.6)	2 (22.2)	Ref
Household structure			
Living alone	6 (10.3)	5 (55.6)	19.37 (2.37, 158.28)
Living with people (couple/couple and kid/Other^a^)	52 (89.6)	4 (44.4)	Ref
Medical condition			
No	15 (25.9)	6 (66.7)	Ref
Yes	43 (74.1)	3 (33.3)	0.11 (0.02, 0.77)

Abbreviations: AOR, adjusted odds ratio; CI, confidence interval; IBD, inflammatory bowel disease; Ref, references.

Other include shared accommodation.

Interview participants commented about sourcing IBD medications from a pharmacy after obtaining prescriptions for their medications from a GP but did not specifically mention whether or not they took the medication as prescribed. From the responses (Supplementary Data 10, which describes data and inferences of quantitative and qualitative analysis), it appeared that participants’ understanding was that their medication is not optional; a non-negotiable part of the management of their IBD and for 1 participant, commencing a new medication had led to improvements in the management of their IBD. Patients are generally prescribed corticosteroids by specialists and GPs as part of self-management of IBD flares whether that be during their flares or in provision for any episodes of flares. Some participants mentioned their reluctance in using corticosteroids, highlighting a degree of medication nonadherence. They avoided using corticosteroids unless it was their only option to circumvent going to the hospital.

### Factors Associated With Health-Related QoL in Patients With IBD

There were no significant differences in any of the QoL scores between UC and CD participants (Supplementary Data 11, which reports factors associated with QoL). With IBD medication-related side effects (*P* = .02), the odds were not significantly higher in participants that suffered medication-related side effects (AOR = 2.76, 95% CI = 0.79, 9.50). Similarly, the odds were higher in individuals who reported having an IBD-related complication (AOR = 2.94, 95% CI = 1.06, 8.14; *P* = .04) when compared to those who did not. QoL scores differed among the participants based on gender (*P* = .04), where the odds of poor QoL (AOR = 1.54, 95% CI = 0.44, 5.35) was higher among male participants. The odds of poor QoL were higher (AOR = 13.24, 95% CI = 0.83, 210.8) among participants taking oral immunosuppressants/prednisone, and almost 3 times higher in those on alternative therapies (AOR = 2.85, 95% CI = 0.58, 14.12), but these findings were not significant (Table 2).

**Table 2.** A multivariable logistic regression of factors associated in quality of life.

Variables	Better QoL *n* (%)	Poor QoL *n* (%)	AOR (95% CI)
Age at the time of IBD diagnosis (years)			
<30	15 (36.6)	16 (61.5)	Ref
≥30	26 (63.4)	10 (38.5)	0.39 (0.10, 1.52)
Gender			
Female	25 (61.0)	9 (34.6)	Ref
Male	16 (39.0)	17 (65.4)	1.54 (0.44, 5.35)
Current management of IBD			
Injectable/biologics	15 (37.5)	7 (26.9)	Ref
Oral immunosuppressant/prednisone	1 (2.5)	7 (26.9)	13.24 (0.83, 210.81)
Aminosalicylates	16 (40.0)	5 (19.3)	1.13 (0.20, 6.29)
Alternative therapies	8 (20.0)	7 (26.9)	2.85 (0.58, 14.12)
Side effect from IBD medications			
No/unsure	30 (73.2)	10 (43.5)	Ref
Yes	11 (26.8)	13 (56.5)	2.76 (0.79, 9.50)
Complications associated with IBD			
Yes	13 (31.7)	15 (57.7)	3.16 (0.89, 11.19)
No	28 (68.3)	11 (42.3)	Ref

Abbreviations: AOR, adjusted odds ratio; CI, confidence interval; IBD, inflammatory bowel disease; QoL, quality of life; Ref, references.

The key QoL factors identified from participant interviews were the absence of IBD (symptoms/flares), “care factor,” and support. Participants considered good QoL to involve the ability to function normally; life outside of their IBD. Several participants indicated being in remission and without having any IBD symptoms was what they considered ideal for living with IBD. Having IBD symptoms presented noteworthy challenges to participants’ QoL and several participants indicated that having an interested, caring health professional or individual involved with their care made a difference to their overall management and general wellbeing. For instance:

It is someone who will listen. My specialist is really great, he listens and cares and that makes all the difference for me. I know the difference…I had the first specialist and since having my current specialist, I’ve seen how it is. (Female, aged 34, diagnosed CD)

Most participants reported some benefits of support groups for the IBD population generally, but did not feel support groups suited their personal needs for IBD management or current care. Some participants had a very strong negative view of support groups but there was 1 participant who found that engaging with a support group was helpful to their overall QoL.

### Patients’ Perception of Primary Care Health Professionals in Managing IBD

**Figure 1.**
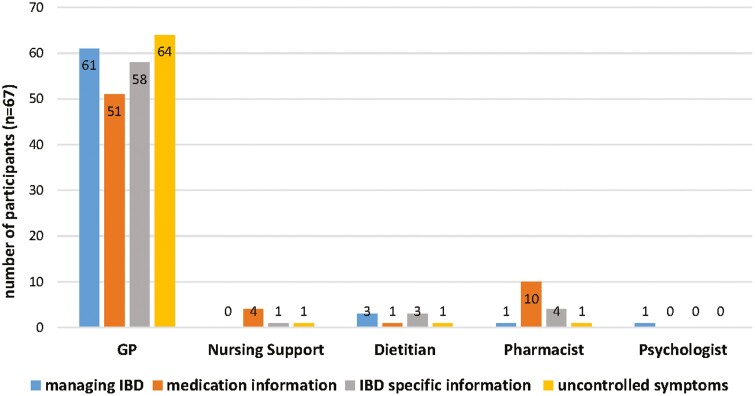
Patients’ perception of primary healthcare professionals who they consider best at managing specific aspects of inflammatory bowel disease (IBD).

**Figure 2.**
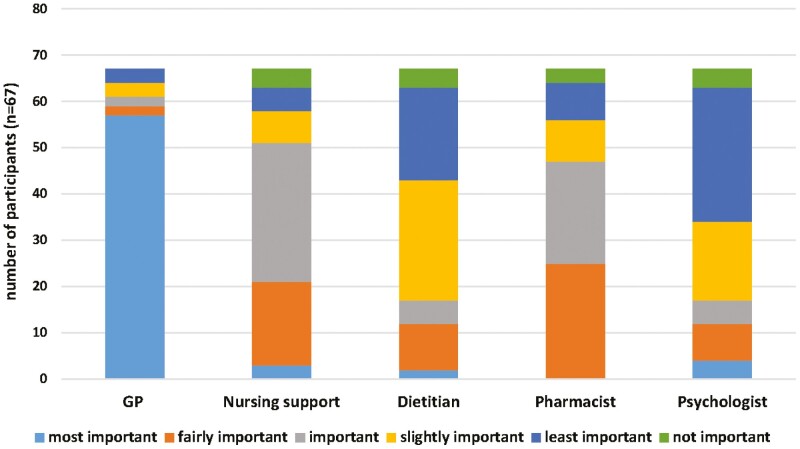
Ranking of patients’ perception on the best-suited healthcare professional involved in managing patients with inflammatory bowel disease (IBD).

Overall, participants perceived their key primary care provider to be the GP for managing all aspects of their IBD (Figure 1). When asked to select one of the best-suited primary healthcare professionals, 61 (91%) of participants preferred GPs to manage their IBD, and 3 (4.5%) a dietitian. Fifty-one (76.1%) participants considered the GP as their preferred source of information about medications, while 10 (14.9%) participants selected pharmacists and 4 participants selected nursing support (this includes nurses practising in primary care). When asked to select their preference about where they would access additional information relating to their IBD, 58 participants (85%) reported a GP as their choice of contact and 4 (6.0%) participants indicated their pharmacist. Sixty-four (95.5%) participants selected their GP as their preferred primary care health professional regarding uncontrolled symptoms. Overall GPs were ranked most important, followed by pharmacists, nursing support, and dietitians, with psychologists ranked as least important (Figure 2).

The qualitative findings regarding health professional preferences of people with IBD support the quantitative results. The majority of the participants reported the importance of GPs as the key primary care provider and the first point of contact for managing all aspects of their IBD. Whilst all of the participants emphasized the important role of their specialists (gastroenterologists) in managing their IBD, some participants considered them as a secondary point of contact after GPs. Some participants used hospital or clinic-based nursing support to facilitate access to information, 1 participant found the nursing support the main source of care for their IBD, while others mentioned the lack of awareness of nursing support or not having utilized them.

The benefits of a psychologist were acknowledged by all participants, however, they reported not having accessed or needing one for managing their IBD. For instance, 1 participant expressed applying certain tools discussed in a session with the psychologist for a family member for their own needs. Several participants viewed dietitian services unfavorably and felt dietitians lacked adequate knowledge and ability to tailor information to their IBD-specific needs. Interestingly, participants who also had diabetes seemed to be aware of the benefits of utilizing a dietitian for managing diabetes. Nevertheless, they did not see the benefits in relation to their IBD. In contrast, participants with no engagement with a dietitian appeared to be more receptive to exploring the benefits of utilizing one in their care. Participants identified other healthcare professionals that contribute to their care that included physiotherapists, naturopaths, exercise physiologists, and pathologists.

The idea of having access to an IBD educator that was easily accessible in primary care was supported by all participants. Participants who also have diabetes readily conceptualized the benefit of a diabetes educator equivalent for IBD and some participants also expressed the availability of such a specialized service could allow for timely access that would optimize the management of their IBD. For example:

Yes, that would be a good service to have one. Access to one should be available and it is surprising that we don’t already have one. (Male, aged 56, diagnosed CD)

### Patients’ Perception Toward Pharmacists’ Role in Managing IBD

Participants’ overall degree of agreement with statements about pharmacists’ expertise and shared decision-making in IBD management was 54.4% (CI 95%; interquartile range: 41.8, 66.5%). Participants’ current age (*P* = .03) and the type of IBD (*P* = .02) they were diagnosed with were identified as 2 independent variables associated with their perception toward pharmacists (Supplementary Data 12 describes factors associated with patient perception of the role of pharmacists). The odds of less favorable perception toward pharmacists was significantly higher among participants with CD (AOR = 9.45, 95% CI = 1.57, 56.62) compared to those with UC or indeterminate colitis/unsure about their diagnosis (Table 3).

**Table 3.** A multivariable logistic regression of factors associated with patients' perception of pharmacists.

Variable	Patients’ perception		COR (95% CI)	AOR (95% CI)
	Favorable	Unfavorable		
Current age (years)				
≤40	7 (22.6)	18 (48.6)	3.25 (1.12, 9.38)	3.08 (0.99, 9.57)
>40	24 (77.4)	19 (51.3)	Ref	
Type of IBD				
Crohn’s disease	8 (25.8)	18 (48.6)	10.1 (1.77, 57.91)	9.45 (1.57, 56.62)
Ulcerative colitis	14 (45.2)	17 (45.9)	5.46 (1.01, 29.54)	5.73 (1.00, 32.57)
Indeterminate colitis/unsure	9 (29.0)	2 (5.4)	Ref	

Abbreviations: AOR, adjusted odds ratio; CI, confidence interval; COR, crude odds ratio; IBD, inflammatory bowel disease; Ref, reference.

The role of pharmacists was reflected in the following subthemes from the qualitative analysis: contribution, knowledge, comfort, benefits of pharmacists, and services. Participants commonly expressed a lack of or limited knowledge and limited or no benefits of pharmacists regarding managing their IBD. Furthermore, some participants identified variability in their interactions with a pharmacist and their knowledge of IBD. While many participants identified pharmacists’ contribution as the source of medication, some participants reported pharmacists with more scope related to managing their IBD. Although benefits were reported by many, 1 participant did not see any benefit in the role of pharmacists in managing IBD.

Most of the participants indicated being comfortable engaging with pharmacists regarding their IBD but many were unsure of their role in managing IBD. In addition, participants suggested the availability of vaccination services and other injections such as vitamin B12 and needle exchange programs as essential services that could be accessed through community pharmacies. They also emphasized the need for better awareness of services available, familiarity with IBD, and better availability of IBD medications as important aspects of services that should be available through pharmacies.

## Discussion

This mixed-methods study measured and explored factors associated with IBD-control, medication adherence, QoL, and perceptions that people with IBD have about healthcare professionals. To our knowledge, it is the first to consolidate such findings so that healthcare professionals can better meet the healthcare needs of IBD patients. Quantitative findings identified the age of diagnosis was as a common factor that was associated with nonadherence, poor QoL, and uncontrolled IBD, and mirror already published studies reporting prevalence in patients who were younger at the time of IBD diagnosis.^11^ The presence of other medical conditions and a person’s living arrangements were seen to influence medication nonadherence. Medication-related side effects, IBD-related complications, and sociodemographic variables such as gender were associated with reduced QoL among this cohort. Poor disease control was observed among participants who experienced medication-related side effects and participants with IBD-related complications. Our observation that 85% of participants who were not classified as being in remission reported not needing a change in treatment is instructive, but consistent with the literature.^37^

The qualitative results of this study highlighted the needs of people living with IBD and reiterated the important role of gastroenterologists in managing their IBD, consistently mentioning specialist’s expertise, understanding of IBD and empathy as highly valued attributes. GPs were reported as the first point of contact for any needs arising from their IBD. The most important aspect of IBD management was reported as the care received from healthcare professionals in managing IBD. Patients described “*good IBD care*” as timely access to services, specialists or healthcare professionals who care and understand their needs and experiences. Participants saw potential benefits for other primary healthcare professionals such as psychologists, dietitians, pharmacists, and nursing support, but they reported a limited role in managing their IBD currently and found that these healthcare professionals lacked disease-specific knowledge.

Timely access to healthcare professionals or services increases the likelihood of IBD patients developing coping strategies that enable them to better self-manage their IBD.^3^ Lack of timely access was reported as the main factor that limited use of specialist services mirroring those from “*My IBD Experience*” survey (*n* = 1024),^38^ where 30% of IBD patients with worsening symptoms or disease control waited an average of 14 days to access a specialist.^38^ GPs are accessible in primary care and are considered as the access point for patients to access medications and specialists. Despite this, GPs are not listed as an integral part of an ideal IBD management team in the current Australian IBD Standards.^6,14^ Other published literature in Australia suggests that’s much of the “out-of-hospital” care for patients living with IBD is delivered by GPs.^13^ This is consistent with findings reported in our previous study.^19^

Our cohort ranked psychologists as “*least important*” in managing patients’ IBD. Although they identified the benefits of psychologists, participants had not accessed this service. This suggests that there is a lack of active awareness, identification, and understanding of the potential role of a psychologist in supporting improved QoL and mental health among people living with IBD. Additionally, IBD increases the risk of malnutrition and is associated with debilitating gastrointestinal symptoms, hence, dietitians can play a valuable role in supporting nutritional needs in the management of IBD. This is reflected in our qualitative findings, where patients indicated diet affecting their disease and sought information from a dietitian. One of the key issues identified was the frustrations patients felt due to dietetic information being conflicting and untailored. This demonstrates the desire of people with IBD for dietitians to have greater IBD-specific expertise and an opportunity for the dietetic profession to enhance IBD-specific training and skills.

Pharmacists play an active and important role in managing patients with chronic diseases.^18^ In this study, pharmacists were considered as the source of IBD medications, and were associated with services like vaccinations, vitamin B12 injections, and disposal of injectables used for their treatment. Participants reported that pharmacists lacked sufficient knowledge or familiarity with the disease to contribute substantially to IBD care. People with CD found pharmacists to be of little help with IBD management compared to those with UC or indeterminate colitis, highlighting the need for pharmacists’ knowledge and education skills specific to types of IBD. This reinforces our findings in a previous study that investigated IBD knowledge among Australian pharmacists, which indicated that pharmacists’ knowledge of IBD and its’ management was suboptimal.^19^ IBD patients may account for a relatively low proportion of patients in primary care practice for pharmacists compared to other chronic conditions such as cardiovascular disease, asthma, or diabetes mellitus.^18^ With adequate training and resources, there is an opportunity for pharmacists to support the decision-making and self-management of IBD patients around steroid initiation and use. For pharmacists to be considered more favorably as “first line” IBD health professionals, easy access to practical services and IBD-specific expertise are needed.

Educators in diabetes and asthma have made considerable contributions toward improving patient-related health outcomes, particularly in primary care.^39,40^ These specializations allow for allied healthcare professionals such as pharmacists, dietitians, and psychologists to be trained to provide adequate support to patients living with chronic conditions and help better manage their needs. A potential opportunity for such a role managing IBD patients in primary care may be beneficial and complement the existing services available in secondary and tertiary care settings. The strong support for IBD educator roles in our findings, especially among participants familiar with diabetes educator roles, indicated that such a service may meet some primary healthcare needs of people with IBD. For example, trained IBD educators could support decision-making about initiating and adjusting medications (steroids) and improve self-management. IBD educator services may complement specialized services and bridge service delivery gaps in IBD speciality, knowledge and access; facilitate improved quality of IBD care, and empower patients toward better understanding and management of their IBD and health.

### Limitations

While our findings support and extend on published literature, we acknowledge the relatively small survey sample size, which may have contributed to Type II errors, participation bias, and potential recall bias. The cross-sectional survey design means that causal inference between selected factors and medication nonadherence, QoL, and disease control was not possible. Participants were mainly enrolled from the DHB, located in the Hunter region (New South Wales, Australia), and therefore may not be representative of the entire Australian IBD population. Because the remainder of the sample came from online recruitment from the Crohn’s & Colitis Australia website, it was not possible to report a definitive response rate.

The validated IBD Control Questionnaire was modified, impacting interpretation and validity of the tool. The questions were changed to assess a 4-week period for control rather than 2 weeks because a 2-week period would not have been an adequate representation of patient disease control for the purpose of our study. A question about clinic visits was removed from this section of the survey, as it was collected elsewhere in the survey. Whilst validating the modified version of the IBD Control Questionnaire was not possible, we used Cronbach’s alpha to measure reliability, and showed high internal consistency (0.89).

Another limitation is the lack of data on race/ethnicity and socioeconomic status, which may have influenced the generalizability of findings about the management of IBD to other countries or between cultural groups. Finally, the global COVID-19 pandemic may have limited participant responses via mail. Despite these limitations, the results of this survey provide an insight into the possible associations between selected sociodemographic, psychological, and physical factors that influence key aspects of IBD management including medication adherence, QoL, disease control, and patient's perception of healthcare professionals. The use of sequential mixed method methodology further mitigated limitations, with quantitative and qualitative analysis triangulated to assist in interpretation and representation of data.

## Conclusion

The perceptions of people with IBD provide valuable insight into the respective roles of, and opportunities for, healthcare professionals involved in their care. This study highlights the importance of specialized, multidisciplinary services for people with IBD that are readily available in primary care to complement specialist medical services. Primary healthcare professionals involved in IBD management could focus on improving their understanding of IBD, enhancing IBD-specific educational capability IBD, and delivering patient-centered, individually tailored care to people with IBD. Further research is needed to investigate decision-making around steroid initiation and use, along with the potential for primary healthcare professionals to be trained as IBD educators to address the gaps in care identified by patients. Optimizing opportunities to allow healthcare professionals to detect nonadherence, poor disease, control and deteriorating QoL in individuals who may be at increased risk of developing further complications is likely to lead to better management of IBD.

## Supplementary Material

otab084_suppl_Supplementary_Data_S1

otab084_suppl_Supplementary_Data_S2

otab084_suppl_Supplementary_Data_S3

otab084_suppl_Supplementary_Data_S4

otab084_suppl_Supplementary_Data_S5

otab084_suppl_Supplementary_Data_S6

otab084_suppl_Supplementary_Data_S7

otab084_suppl_Supplementary_Data_S8

otab084_suppl_Supplementary_Data_S9

otab084_suppl_Supplementary_Data_S10

otab084_suppl_Supplementary_Data_S11

otab084_suppl_Supplementary_Data_S12

## Data Availability

The data underlying this article are available in the article and in its Supplementary material.
