# Supplementary material for: Healthcare Needs and Perceptions of People Living With Inflammatory Bowel Disease in Australia: A Mixed-Methods Study
Source: Crohns Colitis 360. 2022 Jan 3;4(1):otab084. doi: 10.1093/crocol/otab084 (PMC9802190; doi:10.1093/crocol/otab084)
Supplement: otab084_suppl_Supplementary_Data_S1 [file otab084_suppl_supplementary_data_s1.pdf]

THE UNIVERSITY OF NEWCASTLE AUSTRALIA

[INSERT SITE SPECIFIC INSTITUTIONAL LOGO]

HMRI

UNIVERSITY OF NEWCASTLE

PATIENT IBD SURVEY

INTRODUCTION

Thank you for taking the time to answer some questions relating investigating the needs of patients and the role of pharmacists in primary care management of Inflammatory Bowel Disease (IBD).

You have been invited to participate in this study because you are aged 18 years and over and have been diagnosed with inflammatory bowel disease (IBD).

The purpose of this study is to examine your experiences as an IBD patient, and to understand your perceptions of the role of pharmacists in managing IBD. Primary care pharmacists refer to pharmacists working in community pharmacy, GP Clinic, Residential Care, Medication Management Review accredited. The outcome of this study is to optimise the provision of IBD care.

This is a PhD study is being conducted by PhD candidate Sharmila Prasad under supervisors Professor Marjorie Walker, Professor Nicholas Talley, Associate Processor Therese Kairuz, and Associate Professor Simon Keely and, at Newcastle University and the Department of Gastroenterology, John Hunter Hospital.

Your participation is voluntary. The online survey should take you 20-25 minutes to complete. By completing this survey, you are providing consent for the information you provide to be used in the study. If you wish to withdraw from the study, you can do so at any time without having to give a reason if you withdraw prior to submitting the survey.

The information you provide is strictly CONFIDENTIAL. Any personal information will be ANONYMOUS and held securely at the Hunter Medical Research Institute. The results of the survey will be discussed/published in peer-reviewed journals and presentations at medical conferences. In any publication, information will be provided in such a way that you cannot be identified.

This research has been granted human ethics approval: 2019/ETH00167. Should you have concerns or complaints about your rights as a participant in this research, or you have a complaint about the manner in which the research is conducted, it may be directed with the reference number 2019/ETH00167, to the Researcher, or, if an independent person is preferred:

Dr Nicole Gerrand,
Manager Research Ethics and Governance Office,
Hunter New England Local Health District,
Locked Bag 1, New Lambton NSW 2305,
Telephone: (02) 49214950,
Email: hnelhd-hrec@health.nsw.gov.au

Survey Instructions

Please take your time to read each question carefully and answer **ALL** of the questions for each section. Only select **ONE** answer for each of the questions.

Section A: Patient Demographics

1. What is your date of birth? ____/____/_____ (day/month/year)

2. What is your gender identity? (Select one)

   - ☐ Female
   - ☐ Male
   - ☐ Prefer not to say

3. Do you have any medical conditions, or are pregnant or breastfeeding?

   - ☐ No
   - ☐ Yes (please specify)

4. What is your country of birth? (Select one)

   - ☐ Australia
   - ☐ Other (please specify)

5. What is your highest level of education? (Select one)

   - ☐ Year 10 or below
   - ☐ High school graduate,
   - ☐ Diploma level and Advanced Diploma
   - ☐ Bachelor’s degree
   - ☐ Post-graduate degree

6. What is your current employment status? (Select one)

   - ☐ Employed (full-time/part-time)
   - ☐ Self-employed
   - ☐ Unemployed and looking for work
   - ☐ Unemployed but not currently looking for work
   - ☐ A homemaker
   - ☐ A student
   - ☐ Retired
   - ☐ Unable to work

7. What is your current home postcode? (Please specify)

8. Please indicate the structure that best defines your household: (Select one)

   - ☐ Person living alone
   - ☐ Couple only
   - ☐ Couple with children
   - ☐ Other

Section B: Patient History

1. At what age were you diagnosed with IBD? ___________ (age in years)
2. What type of Inflammatory Bowel Disease do you have? (Select one)
   - ☐ Crohn's disease (CD)
   - ☐ Indeterminate colitis
   - ☐ Ulcerative colitis (UC)
   - ☐ I don't know/unsure
3. What were your main symptoms at the time of your diagnosis of IBD? (Please specify)

4. Do you have (or ever had) any extra-intestinal symptoms relating to your IBD (mouth ulcers, rashes, eye symptoms, arthritis etc.)? (Select one)
   - ☐ YES (please specify)
   - ☐ NO
   - ☐ NOT SURE
5. Do you have any complications associated with your IBD (bowel obstruction, perforations, strictures, fistulas)? (Select one)
   - ☐ YES (please specify)
   - ☐ NO
   - ☐ NOT SURE
6. At any time in the past 12 months, was your IBD worse or out of control (relapse)? (Select one)
   - ☐ YES
   - ☐ NO
7. In the past 12 months, have you been admitted to hospital due to your IBD? (Select one)
   - ☐ YES (number of times)
   - ☐ NO
   - ☐ NOT SURE
8. In the past 2 years, have you had surgery due to your IBD? (Select one)
   - ☐ YES ☐ NO ☐ UNSURE
9. Which of the following best describes your smoking history? (Select one)
   - ☐ Currently smoking
   - ☐ Ex-smoker
   - ☐ Never smoked
10. Which of the following best describes your current management for IBD? (Select one)
    - ☐ Injectables/biologics e.g. Remicade, Humira, Symponi, Entyvio (please specify)
    - ☐ Oral immunosuppressants e.g. Imuran, Neoral, Methoblastin (please specify)
    - ☐ Aminosalicylates e.g. Mesasal/Pentasa/Salofalk, Pyralin/Salazopyrin, Dipentum, Colazide (please specify)
    - ☐ Prednisone/Prednisolone (not mutually exclusive)
    - ☐ Alternative therapies (please specify)
    - ☐ No therapies

11. Have you ever had any side effects from any of your IBD medications? (if so, please specify which medication and what happened)
    - ☐ YES (please specify)
    - ☐ NO
    - ☐ NOT SURE

12. What is the main adverse effect of IBD on your life and why? (Please specify)

Section C: Patient IBD Control Assessment

(Modified version of the validated IBD Control Questionnaire – 10 items)

1. Do you believe that: (Select one for each of the questions)	YES	NO	UNSURE
a. Your IBD has been well controlled in the past 4 weeks?	☐	☐	☐
b. Your current treatment is useful in controlling your IBD?	☐	☐	☐

2. Over the past 4 weeks, have your bowel symptoms been? (Select one)

☐ Getting worse ☐ Getting better ☐ Not changed

3. In the past 4 weeks, did you: (Select one for each of the questions)	YES	NO	UNSURE
a. Miss any planned activities because of your IBD?	☐	☐	☐
b. Wake up at night because of symptoms of IBD?	☐	☐	☐
c. Suffer from significant pain or discomfort?	☐	☐	☐
d. Often (> 50% of times) feel lacking in energy (fatigued)?	☐	☐	☐
e. Feel anxious or depressed because of your IBD?	☐	☐	☐
f. Think you needed a change to your treatment?	☐	☐	☐

4. How would you rate your OVERALL control of your IBD during the past 4 weeks? (Select one)
    - ☐ Not controlled at all
    - ☐ Poorly controlled
    - ☐ Somewhat controlled
    - ☐ Well controlled
    - ☐ Completely controlled

Section D: Patient IBD Quality of Life Assessment

QUALITY OF LIFE IN SHORT INFLAMMATORY BOWEL DISEASE QUESTIONNAIRE (SIBDQ) ©

INSTRUCTIONS FOR SELF-COMPLETED SIBDQ

This questionnaire is designed to measure the effects of your inflammatory bowel disease on your daily function and quality of life. You will be asked about symptoms you have been having as a result of your bowel disease, the way you have been feeling in general, and how your mood has been.

On this questionnaire there are 10 questions. Each question has a graded response numbered from 1 to 7. Please read each question carefully and select the number which best describes how you have been feeling in the past 2 weeks.

EXAMPLE

How often have you felt unwell as a result of your bowel problems in the past 2 weeks?

1. ALWAYS
2. ALMOST ALWAYS
3. MANY TIMES
4. SOMETIMES
5. RARELY
6. VERY RARELY
7. NEVER

If you are having trouble understanding a question, STOP for a moment!
Think about what the question means to you. How is this activity or issue affected by your bowel problem? Then answer the question as best as you can. You will have the chance to ask the research assistant questions after completing the questionnaire. This takes only a few minutes to complete.

1. How often has the feeling of fatigue or of being tired and worn out been a problem for you during the last 2 weeks? Please indicate how often the feeling of fatigue or tiredness has been a problem for you during the last 2 weeks by picking one of the options from
   - ☐ ALWAYS
   - ☐ ALMOST ALWAYS
   - ☐ MANY TIMES
   - ☐ SOMETIMES
   - ☐ RARELY
   - ☐ VERY RARELY
   - ☐ NEVER
2. How often during the last 2 weeks have you had to delay or cancel a social engagement because of your bowel problem? Please choose an option from
   - ☐ ALWAYS
   - ☐ ALMOST ALWAYS
   - ☐ MANY TIMES
   - ☐ SOMETIMES
   - ☐ RARELY
   - ☐ VERY RARELY
   - ☐ NEVER
3. How much difficulty have you had, as a result of your bowel problems, doing leisure or sports activities you would have liked to have done during the last 2 weeks? Please choose an option from
   - ☐ AN EXTREME AMOUNT OF DIFFICULTY; ACTIVITIES MADE IMPOSSIBLE
   - ☐ A LOT OF DIFFICULTY
   - ☐ A FAIR BIT OF DIFFICULTY
   - ☐ AN AVERAGE AMOUNT OF DIFFICULTY
   - ☐ A LITTLE DIFFICULTY
   - ☐ HARDLY ANY DIFFICULTY

- ☐ NO DIFFICULTY; THE BOWEL PROBLEMS DID NOT LIMIT SPORTS OR LEISURE ACTIVITIES

4. How often during the last 2 weeks have you been troubled by pain in the abdomen? Please choose an option from
   - ☐ ALWAYS
   - ☐ ALMOST ALWAYS
   - ☐ MANY TIMES
   - ☐ SOMETIMES
   - ☐ RARELY
   - ☐ VERY RARELY
   - ☐ NEVER
5. How often during the last 2 weeks have you felt depressed or discouraged? Please choose an option from
   - ☐ ALWAYS
   - ☐ ALMOST ALWAYS
   - ☐ MANY TIMES
   - ☐ SOMETIMES
   - ☐ RARELY
   - ☐ VERY RARELY
   - ☐ NEVER
6. Overall, in the last 2 weeks, how much of a problem have you had with passing large amounts of gas? Please choose an option from
   - ☐ EXTREMELY PROBLEMATIC
   - ☐ VERY PROBLEMATIC
   - ☐ SOMEWHAT PROBLEMATIC
   - ☐ SLIGHTLY PROBLEMATIC
   - ☐ VERY SLIGHTLY PROBLEMATIC
   - ☐ ALMOST NOT PROBLEMATIC
   - ☐ NOT PROBLEMATIC
7. Overall, in the last 2 weeks, how much of a problem have you had maintaining or getting to, the weight you would like to be at? Please choose an option from
   - ☐ EXTREMELY PROBLEMATIC
   - ☐ VERY PROBLEMATIC
   - ☐ SOMEWHAT PROBLEMATIC
   - ☐ SLIGHTLY PROBLEMATIC
   - ☐ VERY SLIGHTLY PROBLEMATIC
   - ☐ ALMOST NOT PROBLEMATIC
   - ☐ NOT PROBLEMATIC
8. How often during the last 2 weeks have you felt relaxed and free of tension? Please choose an option from
   - ☐ ALWAYS
   - ☐ ALMOST ALWAYS
   - ☐ MANY TIMES
   - ☐ SOMETIMES
   - ☐ RARELY
   - ☐ VERY RARELY
   - ☐ NEVER
9. How much of the time during the last 2 weeks have you been troubled by a feeling of having to go to the toilet even though your bowels were empty? Please choose an option from
   - ☐ ALWAYS
   - ☐ ALMOST ALWAYS
   - ☐ MANY TIMES
   - ☐ SOMETIMES
   - ☐ RARELY
   - ☐ VERY RARELY
   - ☐ NEVER
10. How much of the time during the last 2 weeks have you felt angry as a result of your bowel problem? Please choose an option from
    - ☐ ALWAYS
    - ☐ ALMOST ALWAYS
    - ☐ MANY TIMES
    - ☐ SOMETIMES
    - ☐ RARELY
    - ☐ VERY RARELY
    - ☐ NEVER

Copyright © 1989 McMaster University, Hamilton, Ontario, Canada The Inflammatory Bowel Disease Questionnaire (IBDQ), authored by Dr. Jan Irvine et al, is the copyright of McMaster University (Copyright ©1989, McMaster University). The IBDQ has been provided under license from McMaster University and must not be copied, distributed or used in any way without the prior written consent of McMaster University.

Contact the McMaster Industry Liaison Office at McMaster University, email: milo@mcmaster.ca for licensing details.

Section E: Patient Medication Adherence Assessment
(Please take your time to read each question carefully and answer **ALL** of the questions)

1. Do you take your IBD medication as prescribed by your GP/specialist? (Select one)
   - ☐ YES
   - ☐ NO (please give a reason)

2. Do you feel confident that your current IBD treatment plan will help your condition? (Select one)
   - ☐ YES
   - ☐ NO (please give a reason)

3. At times, I forget to take my IBD medications. (Please indicate how much you agree or disagree with the this statement)
   - ☐ Strongly agree
   - ☐ Agree
   - ☐ Neutral
   - ☐ Disagree
   - ☐ Strongly Disagree

4. Have you skipped or stopped taking your IBD medications because you didn't think it was working? (Select one)
   - ☐ YES (please give a reason)
   - ☐ NO

5. Have you skipped or stopped taking your IBD medications because it made you feel bad? (Select one)
   - ☐ YES (please give a reason)
   - ☐ NO

6. Do you stop taking your IBD medications when you feel better or when your IBD is under control? (Select one)
   - ☐ Never
   - ☐ Rarely
   - ☐ Sometimes
   - ☐ Most of the time
   - ☐ Always

7. Do you change the dose of your IBD medication to suit your needs (like taking more or less than the prescribed dose by your Doctor/Specialist)? (Select one)
   - ☐ Never
   - ☐ Rarely
   - ☐ Sometimes
   - ☐ Most of the time
   - ☐ Always

8. I feel taking my IBD medications more than once a day is inconvenient? (Please indicate how much you agree or disagree with this statement)
   - ☐ Strongly agree
   - ☐ Agree
   - ☐ Neutral
   - ☐ Disagree
   - ☐ Strongly Disagree

9. Have you run out of your IBD medications because you don't get repeats from your Doctor/Specialist on time? (Select one)
   - ☐ Never
   - ☐ Rarely
   - ☐ Sometimes
   - ☐ Most of the time
   - ☐ Always

10. Were you explained the benefits of your current IBD treatment plan by your Doctor/Specialist/Pharmacist? (Select one)
    - ☐ YES
    - ☐ NO

Section F: Patient Perception of Pharmacist Role in IBD Management

(Please indicate how much you agree or disagree with the following statement)

1. The pharmacist understands your condition and treatment.

☐ Strongly agree ☐ Agree ☐ Somewhat agree ☐ Disagree ☐ Strongly disagree

2. The pharmacist is able to provide relevant information about your condition and treatment.

☐ Strongly agree ☐ Agree ☐ Somewhat agree ☐ Disagree ☐ Strongly disagree

3. The pharmacist involves you in decisions about your care.

☐ Strongly agree ☐ Agree ☐ Somewhat agree ☐ Disagree ☐ Strongly disagree

4. The pharmacist is able to provide additional support in managing your condition and treatment.

☐ Strongly agree ☐ Agree ☐ Somewhat agree ☐ Disagree ☐ Strongly disagree

5. The pharmacist is able to address your needs regarding your condition and treatment.

☐ Strongly agree ☐ Agree ☐ Somewhat agree ☐ Disagree ☐ Strongly disagree

For the following questions, other than the Gastroenterologist, which of the listed healthcare professionals are best at managing your IBD. (Please select **ONE** of the options provided for each of the questions)

1. Who would you go to for information about managing your IBD?

☐ GP ☐ Nursing Support ☐ Dietitian ☐ Pharmacist ☐ Psychologist

2. Who would you go to for information about the medications for your IBD?

☐ GP ☐ Nursing Support ☐ Dietitian ☐ Pharmacist ☐ Psychologist

3. Who would you go to for information about additional information about IBD?

☐ GP ☐ Nursing Support ☐ Dietitian ☐ Pharmacist ☐ Psychologist

4. Who do you see when your symptoms are not well controlled?

☐ GP ☐ Nursing Support ☐ Dietitian ☐ Pharmacist ☐ Psychologist

5. Please **rank** from 1 to 5 based on how important you think each of the healthcare professionals are in managing all aspects of your IBD (1 being the most important to 5 being least important).

GP ______ Nursing Support ______ Dietitian ______ Pharmacist ______ Psychologist ______

*** (Nursing support includes IBD nurses, practice nurses, nurse practitioners)*

Thank you for completing this survey.
