# Supplementary material for: Healthcare Needs and Perceptions of People Living With Inflammatory Bowel Disease in Australia: A Mixed-Methods Study
Source: Crohns Colitis 360. 2022 Jan 3;4(1):otab084. doi: 10.1093/crocol/otab084 (PMC9802190; doi:10.1093/crocol/otab084)
Supplement: otab084_suppl_Supplementary_Data_S3 [file otab084_suppl_supplementary_data_s3.docx]

**Supplementary Information 3 – Topic guide for semi-structured qualitative interview**

Thank you so much for joining me today to talk about how your IBD is being managed. To introduce myself, I’m Sharmila Prasad a PhD student being supervised by Professor Marjorie Walker. I’m also a qualified pharmacist. My research is focused on the care of people living with IBD and how pharmacists, as part of the healthcare team, can help better support people to manage their IBD.

Today, I want to give you the opportunity to share your views about your IBD care and about the role pharmacists’ play in the management of IBD. You recently answered a survey called the ‘Patient IBD Survey’ that asked you about your perception of pharmacists and other healthcare professionals in the community. Today is an opportunity for me to develop an even better understanding of your experiences and views about your IBD care, and the role pharmacists might be able to play in providing this care.

You may be wondering why we are interested in this. Well it’s because, as far as we are aware, there aren’t any specific resources available to guide pharmacists about ‘what’ to provide for people living with IBD or ‘how’ to provide care. So, by sharing your experiences and views with me, you will help us develop suitable resources so that pharmacists can better meet your care needs. Does that make sense?

Before we start the interview, do you have any questions about the research or about the interview process?

Are you happy for me to start the recording now?

****************************Interview Topic Guide *****************************

Now, I am going to ask some questions to prompt our conversations about how your IBD is being managed. There are no right or wrong answers to these questions and today, I am interested in listening about your experiences and views from your perspective.

**Participants’ experience of IBD**

1. From your perspective, can you describe what you think of when I say ‘IBD Management’?

Prompt questions:

- In your opinion, what do you think ‘IBD Management’ generally involves?
- Can you give me an example of IBD Management? i.e. services, professionals, treatments, activities.

1. Who do you go to most of the time about caring for your IBD?
   - 1. Probes:
        1. Gastroenterologist (Specialist)
        2. GP
        3. Pharmacist
        4. Nursing support (IBD Nurse)
        5. Psychologist/Psychiatrist
        6. Dietitian
        7. Other (please explain)

Prompt questions:

- What role do these professions have in caring for your IBD? (prompt for those roles not mentioned in response to Q2)

1. When you experience a flare-up of IBD symptoms, what do you normally do?

Prompt questions:

- Where to do go for help when you have a flare-up of IBD symptoms?
- Who do to seek for help when you have a flare-up of IBD symptoms?

1. What does *good* IBD care look like for yourself?

Prompt questions: Can you provide an example of a time you experienced good IBD care?

**Participants’ perception of Pharmacists**

1. ***(Linked to Q2)*** Earlier I asked about whether a pharmacist was part of your IBD care team. Based on your own experience, what role, if any, does your pharmacist play in the management of your IBD?

Prompt questions:

- In what ways has a pharmacist contributed to your IBD care or management?
- How knowledgeable do you find the pharmacist?
- Do you believe that a pharmacist can help you in managing your IBD or provide the care you need? (Could you please explain more on this)
- If not, could you please explain why the pharmacist does not play a role in the management of your IBD?

1. How comfortable are you with discussing about your IBD with your/the pharmacist?

Prompt questions:

- Based on your experience with the pharmacist:
  1. What do you think is working well for you with the care you receive from your/the pharmacist?
  2. Are there any aspects of care not working so well for you?
  3. If so, how can the care you receive from the pharmacist be improved?

1. For people with diabetes, in a pharmacy, there is access to services such as blood sugar monitoring. What sort of services would you like to see be available to you for your IBD (through a pharmacy)?

**Conclusion**:

Is there anything we haven’t spoken about today that you would like to talk to me about before we conclude the interview?

Are there any questions that you think I should have asked you and that I should ask other people?

Thank you for your time and contributions to this study. If you want to contact us for any reason, please don’t hesitate.
