# Supplementary material for: Healthcare Needs and Perceptions of People Living With Inflammatory Bowel Disease in Australia: A Mixed-Methods Study
Source: Crohns Colitis 360. 2022 Jan 3;4(1):otab084. doi: 10.1093/crocol/otab084 (PMC9802190; doi:10.1093/crocol/otab084)
Supplement: otab084_suppl_Supplementary_Data_S4 [file otab084_suppl_supplementary_data_s4.docx]

**Supplementary Information 4** – Framework analysis methods used for qualitative data analysis, including a-priori topics, themes and subthemes that were added to the framework during analysis.

| **Interview guide questions** | **A-priori topic areas** | **Theme and sub theme refinement** | **Example supporting quote** |
| --- | --- | --- | --- |
| From your perspective, can you describe what you think of when I say ‘IBD Management’?  (what and who might this involve) | Disease control | Disease control   - type of care - shared care versus self-care - involvement of GP and specialist - medical care (added) - timing - availability of medical care - immediate care: 24– 48 hours (added) - accessibility - easily accessible IBD services - access to specialist (added) - knowledge - IBD knowledge and guidance - IBD management experience - IBD confidence and expertise (added) - communication - interactions with specialist, GP, allied health and nursing - Treatment expectation (added) - patient education (added) | *I mostly manage it myself. I will do what the specialist or my doctor has told me and if that doesn’t work then I would go to the pharmacist. And if that doesn’t work then I would go to my GP.*  *It is access to specialists and more importantly, it’s the timing of it. You don’t want your specialist to get back to you in a week. A week is a long time for someone with IBD and a lot can go wrong in that time. So timely manner is so important.*  *Having information about my disease. Being guided about what is happening, going to happen, what I am expected to do and how and when…*  *… someone who understands the condition and can relate… have a conversation with you. What you don’t want is being talked at rather than with.* |
|  | Medication adherence |  |  |
| When you experience a flare-up of IBD symptoms, what do you normally do?  Where to do go for help? Who do to seek for help from? | Disease control |  |  |
|  | Quality of life |  |  |
| What does good IBD care look like for yourself? Can you provide an example of a time you experienced good IBD care? | Disease control | Medication adherence   - self-management of steroid - steroid use - flare management (added) - source of medication - medication avoidance/non-adherence - medication management - adherence versus non-adherence (added) | *I am taking these medications and I take it morning and night and have been for like 4 or 5 years now.*  *I really hate taking steroids and mostly try to avoid taking my prednisone if I can.*  *Normally treatment when I have a flare is to go on steroids. But I try not to use them as often. Because I don’t like to.* |
|  | Quality of life |  |  |
| Who do you go to most of the time about caring for your IBD? | Quality of life |  |  |
|  | Perceptions of health care professionals |  |  |
| What role do these professions have in caring for your IBD? Gastroenterologist (Specialist), GP, Pharmacist, Nursing support (IBD Nurse), Psychologist/ Psychiatrist, Dietitian | Perceptions of health care professionals | Quality of life   - absence of symptoms (‘normality’) or remission - bowel motions (added) - no symptoms/symptom control (added) - not having IBD (added) - recovering from symptoms (added) - support - care factor - listens to concerns (added) - responds to requests/helpful (added) - genuinely cares (added) - invested in care (added) - engaged or involved (added) | *For me it’s about recovering from my symptoms… Also so I think being in remission is good IBD care for me. Not having stress. I guess being able to exercise. And staying calm.*  *I get like 1 to 6 bowels in a day. And not having to have them is what I think is IBD management… Not to have no symptoms or discomfort anymore.*  *You might think it weird but just someone who cares. You know I had seven years of not knowing what was happening or why or anything. It was a terrible time for me. I don’t like to talk about it because people think I’m just complaining because I can but they don’t understand.* |
|  | Disease control |  |  |
|  | Medication adherence |  |  |
| Based on your own experience, what role, does your pharmacist play in management of your IBD? | Perceptions of health care professionals |  |  |
| How comfortable are you with discussing about your IBD with your/the pharmacist?  What works well and not so well for you with the care you receive from your/the pharmacist? | Perception of health care professionals | Perceptions of health professionals   - first point of contact/engagement - role of health care professional - IBD management - responsibilities - educator role - advantages/benefits - contribution to IBD management - opportunities (added) - contribution - involvement and engagement of allied health care professionals - involvement/engagement with IBD nurse - engagement/role/impact of respective health care professionals in IBD - knowledge - IBD specific information - Expertise - Variability of IBD expertise (added) - Comfort with health care (added) - benefits of pharmacists - perceived usefulness - access to medication - pharmacy services - vaccinations (added) - Injections (added) - sharps return (added) - specialised services | *My doctor is the first person I go to. If the doctor can, you know, generally work something out. Or if there's something not right; the doctor can sort it.*  *First place I go to is the clinic with the nurses about every 6 monthly. They are very good and everyone should go to them if they are not already.*  *In a nutshell, I have been to see dietitian, and told me I can eat anything. I found them a bit ignorant of things.*  *No I don’t go to the pharmacist for anything for my IBD.*  *IBD educator would be good to have. I don’t know how I would get to see one but I think I would like to.*  *I don’t think they are knowledgeable enough or understand IBD enough to be of any help other than give the medications. But maybe it could be possible that they could play a part or provide better care*  *Also when I have to get me B12 injections. I have to go to the doctor for the script then go to the pharmacy then back to the doctor. Why can’t the pharmacist just give me my injection at the pharmacy/* |
| For people with diabetes, in a pharmacy, there is access to services such as blood sugar monitoring. What sort of services would you like to see be available to you for your IBD (through a pharmacy)? | Medication adherence |  |  |
|  | Perceptions of health care professionals |  |  |
| What role do you think support groups have for your IBD? | Quality of life |  | *Having support is important so I think support groups can be good in some places but they can also not be so good either.*  *Support groups I wouldn’t use unless it was recommended. They talk about complaints too much, a bit depressing.* |

**IBD – Inflammatory bowel disease; GP – General practitioner; B12 - Cobalamin*
