# Supplementary material for: Healthcare Needs and Perceptions of People Living With Inflammatory Bowel Disease in Australia: A Mixed-Methods Study
Source: Crohns Colitis 360. 2022 Jan 3;4(1):otab084. doi: 10.1093/crocol/otab084 (PMC9802190; doi:10.1093/crocol/otab084)
Supplement: otab084_suppl_Supplementary_Data_S6 [file otab084_suppl_supplementary_data_s6.docx]

**Supplementary Data 6** - Participant sociodemographic information

Sixty-seven participants completed the survey, of which, 50 (74.6%) were recruited from the DHB (response rate: 50%) and 17 (25.4%) from the Crohn’s and Colitis Australia website. The demographic and disease characteristics are presented in the tablet provided below. Demographic information was relatively similar between DHB and CCA participants. Thirty-four respondents (50.8%) were female (none reported being pregnant or breastfeeding) and 33 (49.2%) were male. Thirty-one respondents (46.3%) were diagnosed with UC, 26 (38.8%) had CD and 10 (14.9%) reported having indeterminate colitis. Most study participants were born in Australia (n = 57; 85.1%), were over 40 years of age (62.1%), had co-morbidities (68.7%) and never smoked (50.8%). The median (+ Interquartile range) age of patients at the time of the IBD diagnosis was 30 years (+ 24) and at the time of the survey, 54 years (+ 31).

| **Variables** | **Crohn's disease**  $\boldsymbol{n}$**(%)** | **Ulcerative colitis**  $\boldsymbol{n}$**(%)** | **Indeterminate colitis/Unsure**  $\boldsymbol{n}$**(%)** |
| --- | --- | --- | --- |
| **Country of birth** |  |  |  |
| Australia | 20 (76.9) | 28 (90.3) | 9 (90.0) |
| Other* | 6 (23.0) | 3 (9.7) | 1 (10.0) |
| **Current age (years)** |  |  |  |
| ≤40 | 12 (46.1) | 10 (32.3) | 3 (30) |
| >40 | 14 (53.9) | 21 (67.7) | 7 (70.0) |
| **Age at the time of IBD diagnosis (years)** |  |  |  |
| <30 | 16 (61.5) | 11 (35.5) | 4 (40.0) |
| ≥30 | 10 (38.5) | 20 (64.5) | 6 (60.0) |
| **Gender** |  |  |  |
| Female | 15 (57.7) | 15 (48.4) | 4 (40.0) |
| Male | 11 (42.3) | 16 (51.6) | 6 (60.0) |
| **Medical condition/pregnancy/breastfeeding** |  |  |  |
| No | 6 (23.1) | 13 (41.9) | 2 (20.0) |
| Yes | 20 (76.9) | 18 (58.1) | 8 (80.0) |
| **Highest level of education** |  |  |  |
| Year 10 or below/High school graduate | 8 (30.8) | 15 (48.4) | 4 (40.0) |
| Diploma/Bachelor’s/Postgraduate degree | 18 (69.2) | 16 (51.6) | 6 (60.0) |
| **Current employment status** |  |  |  |
| Employed | 11 (42.3) | 15 (48.4) | 4 (40.0) |
| Unemployed / Other** | 15 (57.7) | 16 (51.6) | 6 (60.0) |
| **Household structure** |  |  |  |
| Living alone | 5 (19.2) | 5 (16.1) | 1 (10.0) |
| Living with people (couple/ couple and kid / Other***) | 21 (80.8) | 26 (83.9) | 9 (90.0) |
| **Smoking history** |  |  |  |
| Current smoker | 3 (11.5) | 3 (9.6) | 1 (10.0) |
| Ex-smoker | 8 (30.8) | 14 (45.2) | 4 (40.0) |
| Never smoker | 15 (57.7) | 14 (45.2) | 5 (50.0) |

(*Other* includes New Zealand, England, Scotland, Canada, Austria, United Kingdom, Germany, South Africa, Philippines; other** includes retired, student, homemaker; other*** include shared accommodation).*

The fifty participants that completed the quantitative questionnaire through the DHB were invited to participate in the qualitative study. Nine participants consented to be interviewed with eight participants completing a semi-structured interview; six (75%) were female and two (25%) were male. The average interview duration was 45 minutes. The five topic areas that were the focus of the qualitative study were: medication non-adherence; quality of life; disease control; healthcare professional’s involvement; and role of pharmacists in management and care of people living with IBD (**Supplementary Data 4**, which describes the framework method of the study).
