# Supplementary material for: Healthcare Needs and Perceptions of People Living With Inflammatory Bowel Disease in Australia: A Mixed-Methods Study
Source: Crohns Colitis 360. 2022 Jan 3;4(1):otab084. doi: 10.1093/crocol/otab084 (PMC9802190; doi:10.1093/crocol/otab084)
Supplement: otab084_suppl_Supplementary_Data_S7 [file otab084_suppl_supplementary_data_s7.docx]

**Supplementary Data 7** – Table of IBD-control characteristics of participants

| **Variables** | **Frequency (%)** | | |
| --- | --- | --- | --- |
|  | Yes | No | Unsure |
| IBD has been well controlled in the past 4 weeks. | 14 (69.3) | 13 (21.0) | 6 (9.7) |
| Current treatment is useful in controlling your IBD | 41 (70.7) | 6 (10.3) | 11 (19.0) |
| Miss planned activities because of your IBD | 15 (24.2) | 44 (71.0) | 3 (4.8) |
| Wake up at night because of symptoms of IBD | 28 (44.4) | 35 (55.6) | 0 (0.0) |
| Suffer from significant pain or discomfort | 28 (45.2) | 34 (54.8) | 0 (0.0) |
| Often (> 50% of times) feel lacking in energy (fatigued) | 44 (71.0) | 16 (25.8) | 2 (3.2) |
| Feel anxious or depressed because of your IBD | 26 (42.0) | 35 (56.4) | 1 (1.6) |
| Think you needed a change to your treatment | 13 (21.0) | 45 (72.6) | 4 (6.4) |
