# Supplementary material for: Healthcare Needs and Perceptions of People Living With Inflammatory Bowel Disease in Australia: A Mixed-Methods Study
Source: Crohns Colitis 360. 2022 Jan 3;4(1):otab084. doi: 10.1093/crocol/otab084 (PMC9802190; doi:10.1093/crocol/otab084)
Supplement: otab084_suppl_Supplementary_Data_S8 [file otab084_suppl_supplementary_data_s8.docx]

**Supplementary Data 8** - Association between background characteristics and IBD control of participants

| **Variables** | **Overall IBD Control score*** | ***p*-value (univariate)** |
| --- | --- | --- |
| **Country of birth** |  |  |
| Australia | 63.9 (38.9, 83.3) | 0.31 |
| Other***** | 77.8 (22.2, 88.9) |  |
| **Current age (years)** |  |  |
| ≤ 40 | 66.7 (38.9, 78.9) | 0.46 |
| >40 | 66.6 (38.9, 83.3) |  |
| **Age at the time of IBD diagnosis (years)** |  |  |
| <30 | 50.0 (22.2, 72.2) | **0.01** |
| ≥30 | 72.2 (55.5, 83.3) |  |
| **Gender** |  |  |
| Female | 72.2 (44.4, 83.3) | 0.22 |
| Male | 61.1 (33.3, 73.3) |  |
| **Medical condition** |  |  |
| No | 63.9 (38.9, 81.1) | 0.76 |
| Yes | 72.2 (38.9, 83.3) |  |
| **Highest level of education** |  |  |
| Year 10 or below/High school graduate | 72.2 (44.4, 83.3) | 0.08 |
| Diploma/Bachelor’s/Postgraduate degree | 63.9 (27.7, 75.5) |  |
| **Current employment status** |  |  |
| Employed | 72.2 (38.9, 83.3) | 0.84 |
| Unemployed /other | 61.1 (38.9, 83.3) |  |
| **Household structure** |  |  |
| Living alone | 70 (38.9, 94.4) | 0.56 |
| Living with people (couple/ couple and kid / Other**) | 66.7 (38.9, 83.3) |  |
| **Smoking history** |  |  |
| Current smoker | 78.9 (72.2, 83.3) | 0.51 |
| Ex-smoker | 61.1 (38.9, 83.3) |  |
| Never smoker | 72.2 (38.9, 83.3) |  |
| **Diagnosis** |  |  |
| Crohn’s disease | 72.2 (50.0, 83.3) | 0.13 |
| Ulcerative colitis | 63.9 (38.9, 78.9) |  |
| Indeterminate colitis/Unsure | 36.1 (22.2, 83.3) |  |
| **Extra-intestinal symptom related to IBD** |  |  |
| Yes | 47.2 (22.2, 72.2) | 0.08 |
| No | 72.2 (58.3, 83.3) |  |
| Unsure | 50 (33.3, 83.3) |  |
| **Current management of IBD** |  |  |
| Injectable/biologics | 72.2 (44.4, 83.3) | 1.12 |
| Oral immunosuppressant/prednisone | 36.1 (5.5, 61.1) |  |
| Aminosalicylate | 72.2 (50.0, 88.9) |  |
| Alternative therapies | 50 (27.7, 78.9) |  |
| **Side effect from IBD medications** |  |  |
| No**/**unsure | 78.9 (44.4, 83.3) | **< 0.01** |
| Yes | 42.2 (27.8, 72.2) |  |
| **Complications associated with IBD** |  |  |
| Yes | 47.2 (5.5, 81.1) | **0.03** |
| No | 72.2 (55.5, 83.3) |  |

*^*^ Medians with an interquartile range was used to present the overall IBD control sum score by patients’ characteristics. Comparison between continuous variables were made by using Spearman correlation; comparison between continuous and categorical variables were made by using Kruskal Wallis Test or Mann-Whitney-Wilcoxon Test. (Other* includes New Zealand, England, Scotland, Canada, Austria, United Kingdom, Germany, South Africa, Philippines; other** include retired, student, homemaker; other*** includes shared accommodation).*
