# Supplementary material for: Healthcare Needs and Perceptions of People Living With Inflammatory Bowel Disease in Australia: A Mixed-Methods Study
Source: Crohns Colitis 360. 2022 Jan 3;4(1):otab084. doi: 10.1093/crocol/otab084 (PMC9802190; doi:10.1093/crocol/otab084)
Supplement: otab084_suppl_Supplementary_Data_S9 [file otab084_suppl_supplementary_data_s9.docx]

**Supplementary Data 9** - Association between background characteristics and medication non-adherence

| **Variables** | **Adherence**  $\boldsymbol{n}$**(%)** | **Non-adherence**  $\boldsymbol{n}$**(%)** | ***p*-value^a^**  **(univariate)** |
| --- | --- | --- | --- |
| **Country of birth** |  |  | 0.61 |
| Australia | 50 (86.2) | 7 (77.8) |  |
| Other***** | 8 (13.8) | 2 (22.2) |  |
| **Current age (years)** |  |  |  |
| ≤40 | 22 (37.9) | 3 (33.3) | 0.79 |
| >40 | 36 (62.1) | 6 (66.7) |  |
| **Age at the time of IBD diagnosis (years)** |  |  |  |
| <30 | 24 (41.4) | 7 (77.8) | **0.04** |
| ≥30 | 34 (58.6) | 2 (22.2) |  |
| **Gender** |  |  |  |
| Female | 29 (50) | 5 (55.6) | 1.00 |
| Male | 29 (50) | 4 (44.4) |  |
| **Medical condition/pregnancy/breastfeeding** |  |  |  |
| No | 15 (25.9) | 6 (66.7) | **0.02** |
| Yes | 43 (74.1) | 3 (33.3) |  |
| **Highest level of education** |  |  |  |
| Year 10 or below/High school graduate | 23 (39.7) | 4 (44.4) | 0.79 |
| Diploma/Bachelor’s/Postgraduate degree | 35 (60.34) | 5 (55.6) |  |
| **Current employment status** |  |  |  |
| Employed | 25 (43.1) | 5 (55.6) | 0.50 |
| Unemployed | 33 (56.9) | 4 (44.4) |  |
| Other |  |  |  |
| **Household status** |  |  |  |
| Living alone | 6 (10.3) | 5 (55.6) | **0.004** |
| Living with people (couple/couple and children/Other**) | 52 (89.6) | 4 (44.4) |  |
| **Smoking history** |  |  |  |
| Current smoker | 5 (8.6) | 2 (22.2) | 0.28 |
| Ex-smoker | 22 (37.9) | 4 (44.4) |  |
| Never smoker | 31 (53.5) | 3 (33.3) |  |
| **Diagnosis** |  |  |  |
| Crohn’s disease | 24 (41.4) | 2 (22.2) | 0.50 |
| Ulcerative colitis | 26 (44.8) | 5 (55.6) |  |
| Indeterminate colitis/Unsure | 8 (13.8) | 2 (22.2) |  |
| **Extra-intestinal symptom related to IBD** |  |  |  |
| Yes | 29 (50.0) | 3 (33.3) | 0.64 |
| No | 18 (31.0) | 4 (44.5) |  |
| Unsure | 11 (19.0) | 2 (22.2) |  |
| **Current management of IBD** *(*$n$ *=66)* |  |  |  |
| Injectable/biologics | 20 (35.1) | 2 (22.2) | 0.16 |
| Oral immunosuppressant/prednisone | 6 (10.5) | 2 (22.2) |  |
| Aminosalicylates | 20 (35.1) | 1 (11.1) |  |
| Alternative therapies | 11 (19.3) | 4 (44.4) |  |
| **Side effect from IBD medications (n =64)** |  |  |  |
| Yes | 35 (63.6) | 5 (55.6) | 0.72 |
| No**/**unsure | 20 (36.4) | 4 (44.4) |  |
| **Complications associated with IBD** |  |  |  |
| Yes | 24 (41.4) | 4 (44.4) | 0.86 |
| No | 34 (58.6) | 5 (55.6) |  |

^a^ The p values reflect significant differences between medication non-adherence and patients’ background characteristics resulting from χ2 analysis and Fisher's Exact Test at the level of significance α=0.05. (*Other* includes New Zealand, England, Scotland, Canada, Austria, United Kingdom, Germany, South Africa, Philippines; other** includes retired, student, homemaker; other*** include shared accommodation).*
