# Supplementary material for: Healthcare Needs and Perceptions of People Living With Inflammatory Bowel Disease in Australia: A Mixed-Methods Study
Source: Crohns Colitis 360. 2022 Jan 3;4(1):otab084. doi: 10.1093/crocol/otab084 (PMC9802190; doi:10.1093/crocol/otab084)
Supplement: otab084_suppl_Supplementary_Data_S10 [file otab084_suppl_supplementary_data_s10.docx]

**Supplementary Data 10** - Analysis of quantitative, qualitative data and inferences

| **Topic** | **Quantitative findings** | **Qualitative findings** | **Inference** |
| --- | --- | --- | --- |
| **Disease control** | IBD medication-related side effects (*p = <0.01*), and complications associated with IBD (*p = 0.03*) were independently associated with poor overall IBD control. | When participants were asked about what good IBD care meant to them, answers reported ways improve disease control – ‘H*aving information about my disease. Being guided about what is happening, going to happen, what I am expected to do and how and when’.* | Patient’s lack of awareness and general understanding of their condition can contribute to poor disease control. Not understanding IBD side effects and how to manage them can lead to other issues such as non-adherence. In turn, this can also lead to IBD complications and poor disease control. |
| **Medication adherence** | The odds of medication non-adherence were significantly higher *(AOR=8.04, 95% CI=1.08, 60.10)* among IBD participants aged less than 30 years at the time of IBD diagnosis (*p = 0.04*) and those who lived alone *(AOR=19.37, 95% CI= 2.37, 158.28; p = 0.004)*. | When participants were asked about how they manage IBD flares, answers highlighted avoidance of medication - ‘*I really hate taking steroids and mostly try to avoid taking my prednisone if I can’.* | Younger patients are new to routine medication use. There may be perceived stigma among IBD patients around using corticosteroids. The fear of adverse effects tend to influence patients medication taking habits and lead to intentional non-adherence to their therapy.  Patients that tend to have comorbidities are required to manage with multiple treatments regimes and are more likely to adhere to their therapies. |
|  | While not significant *(AOR=0.1, 95% CI= 0.02, 0.77); p = 0.02)*, the odds of medication non-adherence were 89% less likely among participants who had medical conditions compared with those who had no medical condition. | When asked about what is IBD management, answers indicated that adherence to treatment was considered as part of participants’ acceptance of every day management - ‘*I am taking these medications and I take it morning and night and have been for like 4 or 5 years now’*. |  |
| **Quality of life** | The odds of poor quality of life were 50% more likely among male participants compared to female *(AOR=1.54, 95% CI=0.44, 5.35; p = 0.04)*. | When asked about good IBD care, participant answers included - ‘*I have had …symptoms and it’s quite smelly. I was so embarrassed to talk to anyone about it ...the impact it had on me was quite extensive I have to say’*. | There are gender differences in health care-seeking behaviour. Due to issues surrounding stigma and embarrassment, male patients are less likely to access their healthcare providers for their needs. |
|  | The odds of poor quality of life were not significantly higher among participants who suffered medication-related side effects *(AOR=2.76, 95% CI=0.79, 9.50; p = 0.02)*.  The odds of poor quality of life were higher among participants who suffered complications associated with their IBD *(AOR=2.94, 95% CI=1.06, 8.14; p = 0.04)* when compared to those who did not. | When asked about good IBD care, participant expressed lack of understanding of the effects of the disease on lifestyle as an issue - *‘You know I had seven years of not knowing what was happening or why or anything. It was a terrible time for me’*. | IBD patients report changes to their quality of life throughout the course of their disease. During active disease, they are more likely to report poor quality of life. Even when in remission, IBD patients can continue to experience poor quality of life due to medication side-effects. |
| **Healthcare Professionals involvement** | Overall GPs were ranked most important, followed by pharmacists, nursing support and dietitians, with psychologists ranked as least important. | When participants were asked which primary care providers were part of their care, - ‘*I would go to my GP always as the first person’.*  *‘I would go to my specialist second after my GP’.*  ‘I wouldn’t go back to the dietitian… *I did not find that it fit someone with IBD. Just wasn’t specific enough’.* | Multidisciplinary team approach is an essential part of IBD management, yet patients in primary care rarely engaged with allied health professionals.  Patients who seek help from other healthcare professional (outside of GPs and specialists) feel there is lack of IBD-specific expertise and knowledge among these health professionals. |
| **Role of pharmacists** | The odds of less favourable perception toward pharmacists was significantly higher among participants with Crohn’s disease *(AOR=9.45, 95% CI=1.57, 56.62; p = 0.02)* and not significantly higher for those with ulcerative colitis *(AOR=5.73, 95% CI=1.00, 32.57; p = 0.02)* compared to patients with indeterminate colitis/unsure about their diagnosis | When asked about the role of pharmacists in IBD management, participants indicated minimal engagement - ‘*I don’t go to the pharmacist for anything for my IBD’.*  *‘There is different level of how much they know’.*  *‘The pharmacist haven’t really sort of done anything. I mean, they might say, hey, listen, take this’.* | Pharmacists may be available to IBD patients in primary care, but patients are not aware of any potential benefits to aid in their management of IBD.  Like other healthcare professionals, patients who seek help from a pharmacist feel that there is variability in knowledge among pharmacists.  Pharmacists are seen only as source of medication by IBD patients. |

**IBD – Inflammatory bowel disease; GP – General practitioner.*
