# Supplementary material for: Healthcare Needs and Perceptions of People Living With Inflammatory Bowel Disease in Australia: A Mixed-Methods Study
Source: Crohns Colitis 360. 2022 Jan 3;4(1):otab084. doi: 10.1093/crocol/otab084 (PMC9802190; doi:10.1093/crocol/otab084)
Supplement: otab084_suppl_Supplementary_Data_S11 [file otab084_suppl_supplementary_data_s11.docx]

**Supplementary Data 11** - Association between participant characteristics and quality of life

| **Variables** | **Better QoL^a^**  $\boldsymbol{n}$**(%)** | **Poor QoL**  $\boldsymbol{n}$**(%)** | ***p*-value^b^**  **(univariate)** |
| --- | --- | --- | --- |
| **Country of birth** |  |  |  |
| Australia | 33 (80.5) | 24 (92.3) | 0.29 |
| Other***** | 8 (19.5) | 2 (7.7) |  |
| **Current age (years)** |  |  |  |
| ≤40 | 17 (41.5) | 8 (30.8) | 0.38 |
| >40 | 24 (58.5) | 18 (69.2) |  |
| **Age at the time of IBD diagnosis (years)** |  |  |  |
| <30 | 15 (36.6) | 16 (61.5) | **0.04** |
| ≥30 | 26 (63.4) | 10 (38.5) |  |
| **Gender** |  |  |  |
| Female | 25 (61.0) | 9 (34.6) | **0.04** |
| Male | 16 (39.0) | 17 (65.4) |  |
| **Medical condition/pregnancy/breastfeeding** |  |  |  |
| No | 15 (36.6) | 6 (23.1) | 0.35 |
| Yes | 26 (63.4) | 20 (76.9) |  |
| **Highest level of education** |  |  |  |
| Year 10 or below/High school graduate | 18 (43.9) | 9 (34.6) | 0.45 |
| Diploma/Bachelor’s/Postgraduate degree | 23 (56.1) | 17 (65.4) |  |
| **Current employment status** |  |  |  |
| Employed | 19 (46.3) | 11 (42.3) | 0.75 |
| Unemployed /other | 22 (53.7) | 15 (57.7) |  |
| **Household structure** |  |  |  |
| Living alone | 6 (14.6) | 5 (19.2) | 0.74 |
| Living with people (couple/ couple and kid / Other**) | 35 (85.4) | 21 (80.8) |  |
| **Smoking history** |  |  |  |
| Current smoker | 6 (14.6) | 1 (3.9) | 0.34 |
| Ex-smoker | 14 (34.2) | 12 (46.1) |  |
| Never smoker | 21 (51.2) | 13 (50.0) |  |
| **Diagnosis** |  |  |  |
| Crohn’s disease | 16 (39.0) | 10 (38.5) | 0.29 |
| Ulcerative colitis | 21 (51.2) | 10 (38.5) |  |
| Indeterminate colitis/Unsure | 4 (9.8) | 6 (23.0) |  |
| **Extra-intestinal symptom related to IBD** |  |  |  |
| Yes | 24 (58.6) | 8 (30.8) | 0.08 |
| No | 11 (26.8) | 11 (42.3) |  |
| Unsure | 6 (14.6) | 7 (26.9) |  |
| **Current management of IBD** |  |  |  |
| Injectable/biologics | 15 (37.5) | 7 (26.9) | **0.01** |
| Oral immunosuppressant/prednisone | 1 (2.5) | 7 (26.9) |  |
| Aminosalicylate | 16 (40.0) | 5 (19.3) |  |
| Alternative therapies | 8 (20.0) | 7 (26.9) |  |
| **Side effect from IBD medications** |  |  |  |
| No**/**unsure | 30 (73.2) | 10 (43.5) | **0.02** |
| Yes | 11 (26.8) | 13 (56.5) |  |
| **Complications associated with IBD** |  |  |  |
| Yes | 13 (31.7) | 15 (57.7) | **0.04** |
| No | 28 (68.3) | 11 (42.3) |  |

^a^ Scores >42 is defined as ‘*Better QoL*’. ^b^ The p values reflect significant differences between quality of life scores and patients’ background characteristics resulting from χ2 analysis and Fisher's Exact Test at the level of significance α=0.05. (*Other* includes New Zealand, England, Scotland, Canada, Austria, United Kingdom, Germany, South Africa, Philippines; other** include retired, student, homemaker; other*** includes shared accommodation).*
