# Supplementary material for: Healthcare Needs and Perceptions of People Living With Inflammatory Bowel Disease in Australia: A Mixed-Methods Study
Source: Crohns Colitis 360. 2022 Jan 3;4(1):otab084. doi: 10.1093/crocol/otab084 (PMC9802190; doi:10.1093/crocol/otab084)
Supplement: otab084_suppl_Supplementary_Data_S12 [file otab084_suppl_supplementary_data_s12.docx]

**Supplementary Data 12** - The association between selected background characteristics and patients’ perception toward pharmacists’ role in managing IBD

| **Variables** | **Patients’ perception** | | ***p*-value**  **(univariate)** |
| --- | --- | --- | --- |
|  | **Positive** | **Negative** |  |
| **Country of birth** |  |  |  |
| Australia | 27 (90.0) | 30 (81.1) | 0.31 |
| Other***** | 3 (10) | 7 (18.9) |  |
| **Current age (years)** |  |  |  |
| ≤ 40 | 7 (22.6) | 18 (48.6) | **0.03** |
| >40 | 24 (77.4) | 19 (51.3) |  |
| **Gender** |  |  |  |
| Female | 13 (43.3) | 21 (56.8) | 0.27 |
| Male | 17 (56.7) | 16 (43.2) |  |
| **Medical condition** |  |  |  |
| No | 10 (33.3) | 11 (29.7) | 0.75 |
| Yes | 20 (66.7) | 26 (70.3) |  |
| **Highest level of education** |  |  |  |
| Year 10 or below/High school graduate | 14 (45.2) | 13 (35.1) | 0.40 |
| Diploma/Bachelor’s/Postgraduate degree | 17 (54.8) | 24 (64.9) |  |
| **Current employment status** |  |  |  |
| Employed | 12 (38.7) | 18 (48.7) | 0.41 |
| Unemployed /Other** | 19 (61.3) | 19 (51.3) |  |
| **Household structure** |  |  |  |
| Living alone | 4 (12.9) | 7 (18.9) | 0.50 |
| Living with people (couple/couple and kid/Other***) | 27 (87.1) | 30 (81.1) |  |
| **Smoking history** |  |  |  |
| Current smoker/ Ex-smoker | 15 (50.0) | 18 (48.7) | 0.91 |
| Never smoker | 15 (50.0) | 19 (51.3) |  |
| **Type of IBD** |  |  |  |
| Crohn’s disease | 8 (25.8) | 18 (48.6) | **0.02** |
| Ulcerative colitis | 14 (45.2) | 17 (45.9) |  |
| Indeterminate colitis/Unsure | 9 (29.0) | 2 (5.4) |  |
| **Extra-intestinal symptom related to IBD** |  |  |  |
| Yes | 9 (30.0) | 16 (43.3) | 0.71 |
| No | 16 (53.3) | 13 (35.1) |  |
| Unsure | 5 (16.7) | 8 (21.6) |  |
| **Current management of IBD** |  |  |  |
| Injectable/biologics | 6 (20.7) | 16 (43.2) | 0.26 |
| Oral immunosuppressant/prednisone | 4 (13.8) | 4 (10.8) |  |
| Aminosalicylate | 12 (41.4) | 9 (24.3) |  |
| Alternative therapies | 7 (24.1) | 8 (21.6) |  |
| **Side effect from IBD medications** |  |  |  |
| No**/**unsure | 19 (67.9) | 21 (58.3) | 0.44 |
| Yes | 9 (32.1) | 15 (41.7) |  |
| **Complications associated with IBD** |  |  |  |
| Yes | 11 (35.5) | 17 (46.0) | 0.38 |
| No | 20 (64.5) | 20 (54.0) |  |

(*Other* includes New Zealand, England, Scotland, Canada, Austria, United Kingdom, Germany, South Africa, Philippines; other** include retired, student, homemaker; other*** includes shared accommodation).*
